# Factors Influencing Inappropriate Use of Antibiotics in Infants under 3 Years of Age in Primary Care: A Qualitative Study of the Paediatricians’ Perceptions

**DOI:** 10.3390/antibiotics12040727

**Published:** 2023-04-07

**Authors:** José Arnau-Sánchez, Casimiro Jiménez-Guillén, Manuel Alcaraz-Quiñonero, Juan José Vigueras-Abellán, Beatriz Garnica-Martínez, Juan Francisco Soriano-Ibarra, Gema Martín-Ayala

**Affiliations:** 1 General Directorate of Health Planning, Research, Pharmacy and Citizen Services, Health Counseling of Murcia Region, 30001 Murcia, Spain; 2 Research Group of Murciano Institute of Biosanitary Research, IMIB, 30120 Murcia, Spain; 3 Faculty of Health Sciences, University of Murcia, 30120 Murcia, Spain; 4 National Plan for Antibiotic Resistance (PRAN) in Murcia Region, 30001 Murcia, Spain; 5 Heath Care Center of Yecla, V Health Area, 30510 Murcia, Spain; 6 Regional Paediatric, Murciano Health Service, 30100 Murcia, Spain; 7 Health Care Center of Lorca Sur, III Health Area, 30800 Murcia, Spain

**Keywords:** antibiotics, early infancy, paediatricians, prescription, primary care, qualitative study

## Abstract

Antibiotic consumption in infants of less than three years is higher than average the average consumption for general population. The aim of this study was to explore paediatricians’ opinions regarding factors influencing inappropriate use of antibiotics in early infancy in primary care. A qualitative study based on the grounded theory using convenience sampling was conducted in Murcia Region, Spain. Three focal discussion groups were developed with 25 participants from 9 health areas (HA) of Murcia Region. Paediatricians perceived that health care pressure was an influential factor in the prescribing behaviour, forcing them to prescribe antibiotics for a rapid cure in unjustified circumstances. Participants believed that antibiotic consuming was related to parents’ self-medication due to their perceptions about the curative potential of antibiotics together with facilities to obtain these agents from pharmacies without prescription. The misuse of antibiotics by paediatricians was associated to the lack of education on antibiotic prescription and the limited use of clinical guidelines. Not prescribing an antibiotic in the presence of a potentially severe disease generated more fear than an unnecessary prescription. The clinical interaction asymmetry was more evident, when paediatricians use trapping risk strategies as a mechanism to justify a restrictive prescribing behaviour. The rational model of clinical decision-making in antibiotic prescribing among paediatricians was determined by factors associated with health care management, social awareness and knowledge of the population and pressure of families’ demands. The present findings have contributed to the design and implementation of health interventions in the community for improving awareness of the appropriate use of antibiotics, as well as for a better quality of prescription by peadiatricians.

## 1. Introduction

Antibiotic consumption in infants less than three to five years of age continues to be significantly higher than the average in the general population, being mainly prescribed for treating common paediatric illnesses without clear benefits derived from antibiotic therapy [1,2]. In recent years, however, there has been an increased awareness and recognition of the adverse effects of antibiotic overuse, which has contributed to reducing the rate of antibiotic prescription in paediatric patients [3]. According to data of the European Centre for Disease Prevention and Control (ECDC), the percentage of antibiotic consumption in Spain, both in the hospital and in primary care centers, is one of the highest in comparison with other European countries, increasing from a 15.7 daily dose per 1000 inhabitants in 2012 to a 20.0 daily dose per 1000 inhabitants (DID) in 2021 [4]. As regards antibiotic consumptions in infants under three years of age, there are no official nationwide figures data in Spain [5]. However, there is evidence suggesting a high antibiotic consumption in this age group [6]. The large part of antibiotic consumption in Spain comes from the primary care setting and diagnoses related to upper respiratory tract infections. This research arises from analyzing official data on the consumption of antibiotics in infants under three years of age provided by the Department of Health of the Region of Murcia (Spain) [7]. There was a high consumption of antibiotics in this age group, with a prevalence of prescription of 45.7% in 2019. In relation to this prevalence of antibiotic prescription, it was identified that 61% of children under three years of age consumed at least one antibiotic product in this year. In addition, it was found that 67% of antibiotic prescriptions in children younger than three years old were due to treatment of upper respiratory tract infections. In addition, it was observed a large variability in the use of antibiotics among the nine health areas and their corresponding primary care centers of the Region of Murcia, which was unrelated to socio-sanitary factors. In this respect and based on data of Pharmacy Information System of the Department of Health (FACETA), variability among health areas ranged between 13.06 and 25.21 DID, with substantial differences up to seven times higher between the area with the lowest and the area with the highest prescription rates. For the analysis of variability of antibiotic consumption in children under three years of age, the defined daily dose per 1000 inhabitants was the variable selected, excluding prescriptions made in the private sector, drugs used in the hospital setting and public clerk insurances. Accordingly, to develop possible interventions that will effectively improve the use of antibiotics, it is necessary to gather information and understand paediatricians’ prescribing behaviour [8].

Therefore, a qualitative design was used for this study to explore factors influencing inappropriate use of antibiotics in early infancy from the perspective of the primary care paediatrician.

## 2. Methods

### 2.1. Study Design and Participants

The study was designed in the framework of the grounded theory [9], which involves a cyclic process of data collection and development of a provisional scheme of codification. A constant comparison process was used, with data constantly compared to data that were already gathered and pertinent concepts identified and assigned categories and sub-categories, giving rise to the central theory that explains the phenomenon being studied. Focus group discussions (FGDs) was used to explore paediatricians’ perceptions of antibiotic prescription in the primary care setting. The FGD technique is the best tool for generating interactive discussion and addressing subjective aspects from diverse points of view, something that is difficult to achieve with quantitative methods [10].

A convenience sampling method was used. A network of 25 paediatrician leaders from the Health Care System of the Region of Murcia was built. The criteria to be considered leader were as follows: (i) paediatricians who have the ability to influence on the clinical practice of their colleagues and are aware of the need of designing health strategies to improve the use of antibiotics in the paediatric population.

Participants were primary care paediatricians working in the nine health care areas of the public health care system of the Region of Murcia and were recruited by telephone and e-mails. Professionals involved in health care management activities or working in private centres were excluded. None of the selected participants refused to participate in the study. We sought to ensure a high degree of heterogeneity in the composition of the group in terms of age, gender, years of practice and health areas (HAs) of provenance in order to cover the widest range of opinions. Demographic data are shown in Table 1 and Table S1 (Supplementary Materials).

### 2.2. Data Collection

The participants were gathered at the Hospital Clínico Universitario Reina Sofia in Murcia. We explained what the study consisted of and its aims. Three simultaneous focus groups discussions were developed in different classrooms of the hospital. The interviewers were two women and one man. All of them were experts in conducting health-related qualitative studies. The man had a bachelor degree in anthropology and was expert in qualitative research. None of them had prior knowledge of antibiotics or relationship with the participants before the beginning of the study. The main investigator and the co-authors were not included in the management of health policy in HAs to limit the risk of bias. In order to facilitate conduction of the focal group, moderators followed the model proposed by Seidman [11]. We drew up a script of open-ended questions related to the following aspects: (i) knowledge of antibiotics; (ii) antibiotic resistances; (iii) antibiotic prescriptions; (iv) barriers to make an appropriate use of antibiotics; and (v) strategies to make better use of antibiotics. The FGD guide was developed by the research team, previously pilot-tested with a group of 3 paediatricians from HAs: I, II and IX, who were excluded from the study. The final interview guide is shown in Table S2 (Supplementary Materials).

The duration of group sessions was between 60 and 90 min each, and they were recorded with a video camera and in MP3 format for further transcription and analysis. The sessions came to end, when no more new ideas or contributions forthcame from participants. They were coded by range age and gender (“M” for men and “W”for women), and each group was identified with a serial number (FGD1, FGFD2 and FGD3). At the end of the sessions, a general debate was established, recapitulating essential points of discussion and drawing conclusions. The data collection process was carried out in November 2021.

### 2.3. Analysis

For the analysis of data, the theoretical development proposed by Strauss and Corbin [12] based on the grounded theory, the theoretical sampling and the constant comparative method was used. To ensure credibility, in the first phase, open coding was performed in which fragments of the participants’ discourse were linked to the code labels. Subsequently, we performed focused coding, which consisted of searching the most frequent or significant codes for the development of emerging categories. In the second phase, the provisional categories, establishing their properties and dimensions (axial coding), were elaborated. The development of the coding paradigm facilitated the subsequent establishment of relationships between the categories as part of the selective coding process. In a cross-sectional way, a series of analytical, methodological, theoretical and bibliographic memoranda were generated in order to ensure the traceability of the analysis process [12]. To ensure the reliability of the study, results were illustrated with textual fragments from the transcripts of the paediatricians’ recordings. In order to systematize and provide support to the analytical process, working standards and the MAXQDA 10 software system were used throughout the entire process. Data analysis was performed by an anthropologist and nursing degree (J.A.-S. PhD), a pharmacist (G.M.-A.) and two paediatricians (J.J.V.-A. PhD) and (M.A.-Q. PhD), who are working in primary care. Each FGD was analyzed individually, in the interests of reducing any risk of research bias, and then compared with the other groups. Categories were identified, discussed and agreed with all members of the research team. All members of the research team had regular discussions throughout the collection/analysis of data and the development of the theoretical framework as a means to avoid the influence of any preconceptions towards the dataset and resolve any inconsistencies emerging during the process of data analysis. Any disagreements as regards interpretation were discussed by the researchers and resolved by consensus. In line with the grounded theory approach, data collection stopped, when categories were saturated and no new categories emerged [12]. The Consolidated Criteria Reporting Qualitative Studies (COREQ) was followed during the study process [13] (Table S3 in Supplementary Materials).

### 2.4. Ethical Considerations

The study protocol was approved by the Clinical Research Ethics Committee of Hospital Clínico Universitario Virgen de la Arrixaca (protocol code: 201706-PI). The study was conducted following the ethical standards outlined in the World Medical Association Declaration of Helsinki guideline [14]. Written informed consent was obtained from all paediatricians who agreed to participate in the study (Supplementary Materials: S4). In addition, participants were informed about the study objective, confidentiality of responses and their right to discontinue their participation at any time.

## 3. Results

Of the 25 paediatrician leaders who participated in the study, there were 13 men and 12 women. All of them had between 1 and 35 years of practice (62% of participants had more than 10 years of practice, and 38% of participants had less than 10 years of practice). The main categories and the sub-categories identified are shown in Table 2.

### 3.1. Management and Organisation of Health Care

Most paediatricians perceived health care pressure as an influential factor in their prescribing behaviour being forced to prescribe antibiotics for a fast cure in unjustified circumstances, instead of waiting for the course of the illness. In addition, work overload prevented the ordering of the complementary tests required for diagnosis, causing an atmosphere of uncertainty that makes the appropriate management of antibiotics in primary care difficult.

Lack of availability of antimicrobial resistance in primary care was reported as a barrier to appropriate antibiotic prescription. The majority of professionals believed that the information would help to be aware of the dimensions of the problem of antibiotic resistance in their health care area. Reports of antimicrobial resistance were considered very useful to take decisions regarding the most appropriate antibiotic agent, contributing to improving the quality of prescription in primary care. Some paediatricians, however, considered antibiotic resistance as a problem that affects in-patient care only.

In relation to information connectivity problems in primary care, most professional cited difficulties in sharing relevant data of the clinical condition and clinical course of the child due to the absence of a single electronic health record system. According to the participants, shared health records would allow monitoring of the child’s disease from any geographical point, knowing which professionals are involved in the care of the patient and monitoring of the prescribed antibiotic treatments, contributing to the improvement in the quality of care of the paediatric population.

### 3.2. Community, Knowledge and Social Awareness

Paediatricians perceived that parents’ self-medication to their children was related to over-perception of the curative potential of antibiotics for any infectious process, together with facilities to obtain these drugs from community pharmacies without medical prescription. This habit has been influenced by previous experience of the parents in the management of infectious conditions of their children. For some professionals, easy access to antibiotics may account for difficulties in the population’s awareness of the risk associated with inappropriate antibiotic use and the need of being supervised by the prescribing physician. Moreover, professionals recognised that the population’s education and level of knowledge of antibiotic use was poor, and although some paediatricians offered the families informative leaflets on the management of antibiotics, these interventions are clearly insufficient for general social awareness.

### 3.3. The Family Looking for Antibiotic Treatment

Paediatricians perceived that parents demanded for antibiotic treatment to their children when the infectious process presented with sustained fever. In these circumstances, parents had high expectations because of the desire to alleviate the severity of the symptom and to reduce the duration of disease, despite the fact that the use of antibiotics was appropriate. Some paediatricians reported that family pressure may be so high that it becomes difficult to disregard the wishes and expectations of the parents. Participants also reported that when the paediatrician’s care is perceived as unsatisfactory, parents go to public and private emergency services to seek for antibiotics. These health care centres may become spaces that induce antibiotic prescription in the paediatric population. Some verbatim examples of the aforementioned sub-categories are shown in Table 3.

### 3.4. Difficulties of Antibiotic Prescribing Decisions

Paediatricians believed that primary care physicians have not received specific training for antibiotic prescribing decisions in children. On the other hand, misuse of antibiotics by paediatricians was associated to the lack of continuous education on antibiotic prescription and the limited use of clinical practice guidelines as a tool that facilitates clinical decision-making among prescribers.

Most paediatricians stated that non-prescription of an antibiotic to a child that may suffer from a potentially severe disease generated more fear and insecurity than the consequences of an unnecessary prescription. Doubts regarding prescriptions resulted from previous adverse experiences in the management of antibiotics, triggering a feeling of fear for the course of the medical process. In addition, professionals perceived deferred (delayed) prescriptions as a valuable resource to confront uncertainty. However, this delayed prescription was considered a “double-edged sword” in case of diagnostic uncertainty. Participants felt more secure, when antibiotics were used for treating otitis media and tonsillitis than for other type of infections.

On the other hand, paediatricians considered that if the families do not perceive interest or concern for the child’s health status on the part of the paediatrician, this causes a lack of adherence of the family to the doctor’s indications, resorting to another level of care for antibiotic treatment. This clinical interaction asymmetry was more evident, when paediatricians used trapping risk strategies as a mechanism of control and authority on the families to justify a restrictive prescribing behaviour. Participants, however, believed in a symmetrical relationship in which families received information and perceived the physician as a close and accessible doctor for any problem that may arise. In this respect, communication skills of physicians were considered a useful tool for generating confidence and security in the parents.

## 4. Discussion

Antibiotic prescription is a complex process that involves key aspects of the rational model of clinical decision-making influenced by socioeconomic, organizational, professional and clinical aspects [15,16]. This study has shown a relationship between the insufficient knowledge of the population regarding the adequate use of antibiotics and the inappropriate use of antibiotics by the population. Similar findings have been found in qualitative research [17,18,19,20]. Although numerous qualitative studies have explored factors influencing prescribing behaviour of physicians, there is little information on the perceptions of primary care paediatricians on the inappropriate use of antibiotics in early infancy. Paediatricians perceived that poor knowledge of the population associated with easy access to antibiotics without a prescription in community pharmacies is one of the major concerns for parenteral self-medication to their children and poor social awareness of the risk of inappropriate antibiotic consumption. These findings are consistent with high non-prescription-dispensing rates reported in other studies [21,22,23,24,25]. Despite being illegal, over-the-counter sales of antibiotics still occurs in Spain [24,25,26]. The importance of education campaigns in places frequented by parents and reinforcement of public awareness of the risk of inappropriate use of antibiotics was emphasized by participants, but the effectiveness of these interventions remains unclear [27,28,29]. The present results show that health care management interfered in the professionals’ awareness of antibiotic resistance due to the lack of availability of antimicrobial resistance reports. In agreement with other studies in which antibiotic misperception has been shown to be a major driver of resistance [16], antibiotic resistance was perceived by participants as a problem, but there appeared to have no impact on their daily clinical practice, a circumstance that makes it difficult to improve awareness and compromise for improving attitudes and behaviour of prescribers [16,30,31,32].

In relation to clinical decision-making, the professional’s insecurity in cases of non-definitive diagnoses and anxiety relief of the fear of not prescribing antibiotics due to negative previous experiences [33,34] increased antibiotic prescribing decisions in an attempt to protect children from a potentially severe infection. Participants reported to solve diagnostic uncertainty using rapid diagnostic tests and deferred prescriptions. NICE guideline also recommend a backup (delayed) antimicrobial prescribing guideline when there is clinical uncertainty about whether a condition is self-limiting or is likely to deteriorate [35]. Delayed prescribing is also an effective strategy to reduce prescription in those parents with high expectations on antibiotic therapy. In a systematic review, a meta-analysis of nine randomized controlled studies and one observational cohort study totalling 55,682 patients with respiratory tract infection, delayed prescribing was associated with a similar symptom duration as no antibiotic prescribing and is unlikely to lead to poorer symptom control than immediate antibiotic prescribing [36]. However, some participants considered that delayed antibiotic prescribing would be unsafe in case of diagnostic uncertainty, with a fear of missing serious complications as a significant barrier.

Participants also reported that emergency services are used by parents to receive an antibiotic prescription. It has been found that parents grossly overestimate the benefit of antibiotics in reducing the duration of acute respiratory infections in their children. Emergency department (ED) clinicians frequently face with the combination of diagnostic uncertainty, time pressure and parental demands, and different studies have found that antibiotics is a common intervention in the ED with over one in three antibiotic prescriptions being assessed as inappropriate [37,38,39]. The present study, however, does not provide data on factors affecting prescribing behaviour of clinicians in the ED setting.

The need of improving the paediatricians’ knowledge of using antibiotics was recognised as an important aspect to reduce fear and anxiety, as well to increase confidence in the clinical decision-making process [40,41]. The implementation of educational interventions may well serve to improve antibiotic prescriptions given that physicians’ knowledge and attitudes are potentially modifiable factors [42,43]. The UK Antimicrobial Resistance Strategy and Action Plan 2019–2024 [44] emphasizes the importance of professionals’ education on the use of antibiotics. Decisions on prescribing antibiotics were also compromised by the lack of adherence to clinical practice guidelines.

Although participants perceived a direct relationship between the time dedicated to clinical interaction and the possibility of discussing risks and benefits of treatment options, workload pressure in primary care was an important driver of fast antibiotic prescription to save time and to reduce the frequency of visits of children who had been already attended to. In the Region of Murcia, based on internal data of the Murcia Health Service, the mean number of children assigned to each paediatrcian is 861, and the mean number of paediatric patients attended to per day and per paediatrician is 26.18, doubling that mean in winter due to a large amount of common paediatric illnesses, which occur in that seasonal period.

A large number of participants believed that interventions focused on an asymmetrical clinical interaction contribute to the loss of confidence and safety of parents, increasing the difficulties in the decision-making prescription process of professionals. Similar findings were reported in a recent qualitative study carried out in Spain [18].

Participants also mentioned an interactional resistance of parents to non-prescribing antibiotic behaviour. However, a strategy based on the risks of antibiotic use to prevent prescribing generated a passive attitude on the part of parents to therapeutic indications of the paediatrician, transmitting uncertainty and fear regarding their children’s illness.

The results of the present study have contributed to the design of strategies and interventions for both medical professionals and the community within the framework of the Program for the Responsible Use of Antibiotics in Early Childhood (PURAPI Program) of the Health Department of the Region of Murcia. Regarding medical professionals, seminars are currently being given by pediatricians to address the appropriate use of antibiotics in practice conditions and the management of uncertainty in clinical decision-making in the settings of primary care and hospital and extra-hospital emergency services. This activity aimed to update knowledge and quality of prescription among professionals. In addition, this training activity provides skills for managing fear and insecurity when professionals are faced with decision-making.

In relation to the community, workshops on the appropriate use of antibiotics in the management of the most prevalent diseases in infants (bronchiolitis, fever, colds and acute gastroenteritis) are being carried out. The content of these workshops is compiled in the “Guides for families of diseases in childhood” (available at: https://www.escueladesaludmurcia.es/escuelasalud/cuidarse/pediatria/guiasanticipatorias.jsf accessed on 27 March 2023). This is intended to make the population aware of the importance of the appropriate use of antibiotics. Such awareness would contribute to reducing the demand for antibiotics from families to professionals who carry out their activity both in primary care and in the hospital and extra-hospital emergency services.

Finally, from the Department of Health of the Region of Murcia and the Murcia Health Service, systems are being developed to have available data on paediatric patients attended to at each care level, allowing better access to patient information and, consequently, an improvement in the quality of care.

This study was conducted in primary care, so that the present findings should be cautiously generalized to other health care settings. Although theoretical saturation was reached in the three focal groups, other perspectives or points of view of paediatricians that were not categorized as leaders could be missed. The professional profiles of other professionals (e.g., ED physicians) who are also prescribers of antibiotic therapy in children were not analysed.

The research team considers that this selection constituted a representative sample of paediatricians in the Region of Murcia, given that all the leaders are involved in the patients’ care (not management activities) in the primary care setting. The fact of being considered leaders does not imply extensive knowledge about antimicrobial resistance or quality of prescription in their clinical practice. This circumstance allowed us to explore the phenomenon with the least bias possible. However, it could provide a reduced view of the study phenomenon. Therefore, the results must be interpreted with caution.

On the other hand, the years of experience of the participants have been considered to generate a heterogeneous sample in the constitution of the focus groups, in order to broaden the perspective of the phenomenon among the participants belonging to different generations. However, this variable does not provide relevant information on the influence of the years of practice of professionals in the appropriate use of antibiotics. It would be interesting to carry out further research on this matter in the same study area.

## 5. Conclusions

Based on findings of the present qualitative study, the opinion of paediatricians working in primary health care is that the rational model of clinical decision-making in prescribing paediatricians is influenced by factors associated with health care management, social awareness and knowledge of the population about the risk of the misuse of antibiotics, as well as difficulties for professionals in making decisions about antibiotic use and families’ demands for antibiotic prescription. This study highlights the importance of making professionals aware of the responsible prescription of antibiotics and defining strategies focused on a bidirectional way from the perspective of paediatricians and parents with the support of the health care administration to contribute to changing the socio-cultural paradigm of antibiotic consumption and to obtain sustained effects. Further research is needed involving stakeholders, such as patients and pharmacists, to have deeper knowledge of individual aspects, which influence on the misuse of antibiotics not only from prescribers, but also from the community. This would aid in a more thorough understanding of and response to the factors that drive antibiotic prescription, guiding an effective antibiotic stewardship program. Currently, the results of this research have contributed to implementing health interventions for paediatricians within the framework of the PURAPI program.

## Figures and Tables

**Table 1:** Respondents’ demographic characteristics.

FGD	Number of Participants	Male	Female	Years of Practice	Age Range	Health Area
1	8	4	4	1–35	28–63	I, II, III, VI, VII, VIII and IX
2	8	5	3	1–35	32–59	I, II, III, IV, V, VII, VIII and IX
3	9	5	4	1–35	30–65	I, II, III, IV, V, VI and VII

**Table 2:** Categories and sub-categories emerging from FGDs.

Categories	Sub-Categories
1. Management and organisation of health care	1.1. Health care pressure in primary care consultations 1.2. Lack of availability of antimicrobial resistance reports in primary care 1.3. Information connectivity problems
2. Community, knowledge and social awareness	2.1. Parents’ self-medication to their children 2.2. Poor education of population
3. Difficulties of antibiotic prescribing decisions	3.1. Poor training and updating knowledge of paediatricians 3.2. Fear and insecurity of non-prescription of paediatricians 3.3. Asymmetrical clinical relationship between the paediatrician and the family
4. The family looking for antibiotic treatment	4.1. Family pressure for antibiotic prescription 4.2. The resource of the emergency department

**Table 3:** Verbatims from Sub-Categories.

Sub-Categories	Verbatims
1.1. Health care pressure in primary care	“healthcare pressure, I think, is one of the things that, at least for me, influences me the most in deciding to give antibiotics to children” “When the care pressure is very high we increase the consumption of antibiotics because I can stay for three days but on the fourth day I start to get nervous. I don’t hear anything. I don’t make auscultation, do I give him the antibiotic? maybe it’s a flu that with an analysis, the antibiotic would have been avoided, but when you have 50…, I think that when we go fast and it influences overprescription” (FGD 1-PED3 with less than 10 Y of P (years of practice)).
1.2. Lack of availability of antimicrobial resistance reports	“Yes, we need to have more information (on resistances) closest to the area or region to know the current situation and what should be the focus to avoid it. In other words, we are not really seeing the problem on the front line, I don’t see that we are managing the resistance, but I see that, perhaps, at a global level. We could have information that in our area it is increasing in this or the other and that you were already informed on how to apply it in order to make your own decisions” (FGD3-PED1 with more than 10 Y of P).
1.3. Information connectivity problems	“I don’t see it as a complicated issue [Regarding sharing information]. But for that, yes, we would reach all the places, and there will no longer be other opinions different from the prescription of antibiotics because everyone has the same information. And if the mother says “They sent you this antibiotic in the emergency room, can you write me the prescription?” and I look: “No, no, the last time you went to the emergency room was three weeks ago, and they already sent you an antibiotic.” We have to access to that information” (FGD2-PED7 with less than 10 Y of P).
2.1. Parents’ self-medication to their children	“Yes, there is continuing to be self-medication from the parents. They go to the community pharmacy, and people there gave antibiotics that parents request for their children when they are ill. The general philosophy of the population is that antibiotics solve the problem, so that, they say, go buy the drug in the pharmacy and gave it to my child, and that’s all” (FGD3-PED7 with more than 10 Y of P).
2.2. Poor education of the population	“From what we have said about the information about knowledge, I think it is important that there is more social awareness, that there is more education to the population about what can happen to you if you misuse antibiotics … Education is null here […], they should be given information sheets on the use of antibiotics and more should be done” (FGD1-PED1 with more than 10 Y of P).
3.1. Poor training and updating knowledge of professionals	“We have years of the profession, we have experience, and on top of that we have an academic degree. There are many family doctors who prescribe in the emergency room, and it is not that they do it badly, but rather that their specialization has not been as great as ours with this awareness of young children and the problem of overprescribing” (FGD3-PED2 with more than 10 Y of P).
3.2. Fear and insecurity of non-prescription	“For example, the colleague who practices defensive medicine because he passes the consultation with fear, a fear resulting from a bitter experience, that is, a child who has died of meningitis and that you believe that a timely treatment, that is, the antibiotic that you didn’t give him I would have avoided it” (FGD3-PED7 with more than 10 Y of P).
3.3. Asymmetric clinical interaction between the paediatrician and the family	“I believe that the closeness and degree of trust that they have with you and the communication skills that you have to transmit security are very important. That is very important“ […] what I use a lot to parents is fear. I tell them: “look… if it is a bacterial process that has not yet present symptoms that can sometimes cause meningitis, if we give antibiotics it does not show its face and then there is no one to cure that meningitis and it is even worse”, that is to say that there are strategies of fear that can function […]” (FGD1-PED4 with less than 10 Y of P).
4.1. Family pressure for antibiotic prescription	“There are families that don’t tolerate their children having a fever, that don’t tolerate their children’s illness, and the parents have a really bad time and go looking for the pediatrician, and in the end they come to your office so distressed. That in the end they come to you looking for the antibiotic…, and you are forced to send him the antibiotic and you cannot tell him to wait or send him something else […]”. “Because there are families that you can convince them [regarding the prescription], but there are others who say: “Either I put the antibiotic or let’s go.” “And you don’t get complicated anymore.” (FGD2-PED8 with more than 10 Y of P).
4.2. The resource of the emergency department	“There are children that maybe you have seen them and, since they are not calm [regarding the non-prescription of antibiotics], you tell them no, but then they go to the emergency department or go to the emergency room at the hospital, and there is always someone who puts it on”(FGD1-PED3 with less than 10 Y of P).

## Data Availability

The study data are available from the corresponding author (J.A.-S.) upon request. The data are not publicly available due to European Protection Regulation (GDPR).

## Supplementary Materials

The following supporting information can be downloaded at: https://www.mdpi.com/article/10.3390/antibiotics12040727/s1, Table S1. Respondents’ demographic characteristics; Table S2. FGD guide; Table S3. COREQ checklist; S4. Informed consent.

## Abbreviations

DID	Daily Dose per 1000 inhabitants
FGD	Focus Group Discussion
PED	Paediatrician
ED	Emergency Department
HA	Health Areas
Y of P	Years of Practice
