# Supplementary material for: Factors Influencing Inappropriate Use of Antibiotics in Infants under 3 Years of Age in Primary Care: A Qualitative Study of the Paediatricians’ Perceptions
Source: Antibiotics (Basel). 2023 Apr 7;12(4):727. doi: 10.3390/antibiotics12040727 (PMC10135072; doi:10.3390/antibiotics12040727)
Supplement: Supplementary file 1 [file antibiotics-12-00727-s001.zip › S4. Informed consent.pdf]

INFORMED CONSENT

Dear Participant,

Researchers from the Health Counseling of the Murcia Region are carrying out a research study with the purpose of exploring the antimicrobial stewardship in Primary Healthcare from the pediatrician's perspective.

You are being invited to take part in this research because we feel that your experience as a pediatrician can largely contribute to our understanding and knowledge of health practices among pediatricians in the Primary Healthcare setting. You will take part in a focal group made by 7-8 pediatricians from Primary Healthcare of the Murcia Region. This research has been financed by the health research fund. Its aim is to contribute to the improvement of the quality of antibiotic prescription in primary care. This proposal has been reviewed and approved by the Ethics Committee of Arrixaca Hospital (Murcia Region).

The study is being carried out by a group of physicians, nurses, anthropologists and pharmacists interested in exploring possible factors that influence on the use of antibiotics among pediatricians from Primary Healthcare. The purpose of this meeting is to exchange experiences, opinions and points of view on your daily practice about antibiotic prescribing. Your participation in this research is entirely voluntary. In addition, you may stop participating in the focus group discussion at any time that you wish without your job being affected.

The focal group discussion will be taking place in Reina Sofia Hospital (date) and will last for about one hour. The conversation will be recorded for later analysis of the data, but your name will not appear at any time, and anonymity will be maintained throughout the entire process. The recordings made will be destroyed in 4 or 5 months and we will make the results of this research available to you.

For the confidentiality, the research team will not be sharing information about you to anyone. The information that we collect from this research project will be kept privately and will be used only for research proposed.

If you have any questions, you can ask now or later.

If you agree with aforementioned terms, please sign below

Signature of the Participant

Signature of the Researcher

Date:xxxx

Email:xxxx@xxx
