# Supplementary material for: Factors Influencing Inappropriate Use of Antibiotics in Infants under 3 Years of Age in Primary Care: A Qualitative Study of the Paediatricians’ Perceptions
Source: Antibiotics (Basel). 2023 Apr 7;12(4):727. doi: 10.3390/antibiotics12040727 (PMC10135072; doi:10.3390/antibiotics12040727)
Supplement: Supplementary file 1 [file antibiotics-12-00727-s001.zip › Table S1. Participantí»s demographic characteristics.pdf]

antibiotics

MDPI

Table S1. The Focus group discussion participation and participant's demographic characteristics

**Health Area (HA)**	**Focus Group Discussion (FGD) nº participants**		
**HA**	**FGD1**	**FGD2**	**FGD3**
**HA-I**	2	1	2
**HA-II**	1	1	2
**HA-III**	1	1	1
**HA-IV**		1	1
**HA-V**		1	1
**HA-VI**	1		1
**HA-VII**	1	1	1
**HA-VIII**	1	1	
**HA-IX**	1	1	
**Gender**			
**Female**	4	5	4
**Male**	4	3	5
**Years of Practice**			
**1-5**	1	2	1
**5-10**	2	1	2
**15-20**		1	1
**20-25**	1	1	2
**25-30**	2	2	1
**30-35**	2	1	2
**Age Range**			
	28-63	32-59	30-65
