# Supplementary material for: Factors Influencing Inappropriate Use of Antibiotics in Infants under 3 Years of Age in Primary Care: A Qualitative Study of the Paediatricians’ Perceptions
Source: Antibiotics (Basel). 2023 Apr 7;12(4):727. doi: 10.3390/antibiotics12040727 (PMC10135072; doi:10.3390/antibiotics12040727)
Supplement: Supplementary file 1 [file antibiotics-12-00727-s001.zip › Table S2. FGD Guide.pdf]

Table S2. FGDs GUIDE. Questions for focal discussion groups

**Introduction:**<br>Thank you for coming and also to agree to participate in the study. We are talking to you since you are pediatricians and attend children less than 3 years of age.<br>The aim of this discussion is to explore the antibiotic prescribing trends in the outpatient setting among pediatricians from nine healthcare areas of the Murcia Region.<br>A further objective is to understand factors involved at the time of making a decision regarding the prescription of antibiotics at the time of treating common diseases in children less than 3 years of age, and to find measures about health counseling that me be useful to you while prescribing.<br>For your information this interview will be taped recorded, transcribed verbatim and then analyzed by a team of researchers. All information recorded about you and the discussion will be kept confidential and will be deleted once the purpose of this study will be accomplished. You can withdraw from the study at point of time.<br>▪ Do you have any question to what I have explained until now?<br>▪ Could you please sign the informed consent form?<br>Thank you so much for listening and continuing with us for the discussion<br>▪ Please introduce yourself by telling your name, age, working position, years of practice, and healthcare area from which you come from.<br>▪ I would inform you that a number will be assigned to each of the participants in the discussion to keep a track on order of their speaking while transcribing<br><br>Now starting with de focal group discussion session
**1. What knowledge do you have about the use of antibiotics in your healthcare area?**
**2. What are your thoughts on the latest reports from Counseling of Health of the Murcia Region showing an increase in antibiotic consumption?**
**3. How do you think that pediatricians could make a judicious antibiotic consumption in their daily practice?**
**4. What knowledge do you have about antimicrobial resistance in your healthcare area?**
**5. During a working day, what strategies do you carry out for a responsible use of antibiotics in your practice?**

**6.**	**In your opinion, what factors do you think hinder the proper prescription of antibiotics among professionals in the healthcare area of your practice?**
**7.**	**What do you think could influence your prescription behaviour?**
**8.**	**According to your perception, what factors are taken into account when prescribing antibiotics to young children?**
**9.**	**What source(s) of information could have the greatest impact to reduce antibiotic prescribing?**
**10.**	**According to the latest reports from Counseling of Health of the Murcia Region, what can be done to improve it?**
